# Non-tuberculous mycobacteria and other acid fast bacilli pathogens identification by qPCR and MALDI-ToF MS in tuberculosis-like lesions of slaughtered cattle from Ecuador

**DOI:** 10.3389/fvets.2025.1565066

**Published:** 2025-07-08

**Authors:** Solon Alberto Orlando, Leila Estefania Vera Loor, Joselyn Calderón, Melissa Joseth Carvajal-Capa, Fabrizio Arcos Alcívar, Pablo Torres-Lasso, W. Favián Maza Valle, Oliverio N. Vargas González, Naomi Mora-Jaramillo, Ariana León-Sosa, Ariana Rivera, Angel Sebastián Rodríguez-Pazmiño, Bernardo Castro-Rodriguez, Greta Franco Sotomayor, Sandra Uruchima-Campoverde, Manuel Gonzalez, Jose Manuel Benitez-Medina, Natalia Jimenez-Pizarro, Javier Hermoso de Mendoza, Henry Parra-Vera, Miguel Angel Garcia-Bereguiain

**Affiliations:** ^1^Instituto Nacional de Salud Pública e Investigación, Guayaquil, Ecuador; ^2^Universidad Espíritu Santo, Guayaquil, Ecuador; ^3^Escuela Superior Politécnica Agropecuaria de Manabí, Calceta, Ecuador; ^4^Universidad Católica Santiago de Guayaquil, Guayaquil, Ecuador; ^5^Universidad Ecotec, Guayaquil, Ecuador; ^6^Universidad de Guayaquil, Guayaquil, Ecuador; ^7^Universidad Técnica de Machala, Machala, Ecuador; ^8^One Health Research Group, Universidad de Las Américas, Quito, Ecuador; ^9^Unidad de Patología Infecciosa, Facultad de Veterinaria, Universidad de Extremadura, Cáceres, Spain; ^10^Centro de Investigación en Microbiología, Guayaquil, Ecuador

**Keywords:** bovine tuberculosis, cattle, abbatoirs, MTBC, non-tuberculous mycobacteria, *Nocardia*, *Gordonia*, *Tsukamurella*

## Abstract

**Introduction:**

Mycobacterial infections are caused by the *Mycobacterium tuberculosis* complex (MTBC) but also by non-tuberculous mycobacteria (NTM). While the importance of NTM in opportunistic infections in humans has been gaining attention, surveillance and control programs for cattle health and production remain predominantly focused on bovine tuberculosis (BTB) that it is caused exclusively by MTBC.

**Methods:**

In this study, we conducted a comprehensive inspection of 5,803 cattle carcasses destined for human consumption across 22 abattoirs in five provinces of Ecuador’s coastal region, searching for lesions visually compatible with BTB (BTB-like lesions).

**Results:**

A high prevalence of 13.4% (CI 95%: 12.8–14.6) for BTB-like lesions and 3.1% (CI 95%: 2.6–3.5) for acid-fast bacilli (AFB) presence in lesions was observed. From these lesions, we isolated 48 AFB cultures, 44 of which tested positive for NTM. Strikingly, MTBC was not found in any BTB-like lesion by qPCR. Furthermore, Matrix Assisted Laser Desorption/Ionization Time of Flight Mass Spectrometry (MALDI-ToF MS) identified six NTM species: *M. neoaureum*, *M. fortuitum*, *M. novocastrense*, *M. asiaticum*, *M. duvalii*, and *M. mucogenicum*. Additionally, other AFB opportunistic pathogenic species were identified, including *Tsukamurella paurometabola*, *Gordonia hongkongensis*, and *Nocardia* spp.

**Discussion:**

Considering the potential misdiagnosis of NTM and other AFB species, BTB surveillance and control programs for MTBC should be revised to consider other opportunistic infections with similar clinical output to BTB.

## Introduction

Bovine tuberculosis (BTB) is a chronic disease characterized by the development of tubercle-like lesions in various tissues of cattle. However, it also affects a wide range of domestic and wild animals (1, 2). BTB is only caused by species within the *Mycobacterium tuberculosis* complex (MTBC), with *Mycobacterium bovis* being the main pathogen (3). In addition, other members of the genus *Mycobacterium*, collectively referred to as non-tuberculous mycobacteria (NTM), can also cause granulomatous lesions in cattle that resemble those of tuberculosis (TB) (3–5). Infections caused by either MTBC or some species of NTM like *Mycobacterium avium* complex negatively impact cattle health and productivity and serve as a source of zoonotic transmission of TB and other opportunistic mycobacterial infections to humans (3, 6–8).

While high-income countries in Europe and North America have implemented successful surveillance programs to control BTB, low- and middle-income countries (LMICs) in Africa, Asia, and South America continue to face a significant burden of the disease, which is estimated to influence human TB epidemiology (9, 10). Transmission of MTBC from cattle to humans has been associated with the consumption of raw milk or dairy products, as pasteurization practices are not widely adopted in rural communities in LMICs. In this context, it is estimated that approximately 10–15% of human TB cases may be attributable to *M. bovis* (3, 11, 12).

MTBC infections remain a significant animal health issue for dairy cattle, causing substantial economic losses in Ecuador. However, a comprehensive national BTB prevalence map has yet to be developed. Over the last 20 years, a few scientific studies done in Ecuador have reported BTB prevalence rates ranging from 1.01 to 4.33% for BTB-compatible lesions observed in abattoirs (13–15) and from 4.24 to 7.13% for positive reactions to the intradermal tuberculin test (16, 17). However, these reports were confined to only three cantons (Mejía, Cayambe, and Pelileo) within two provinces (Pichincha and Tungurahua) in the Andean region of Ecuador, with the most recent publication dating back to 2013 (13–17).

In Ecuador, the detection of acid-fast bacilli (AFB) through Ziehl–Neelsen staining of lesions visually compatible with TB obtained during human sputum or cattle necropsy examinations is still used for tuberculosis diagnosis (personal communication to authors). However, this method lacks specificity in distinguishing between MTBC and NTM, leading to potential misdiagnosis of TB either in humans or in cattle (3, 18–21). While the studies addressing BTB caused by MTBC in cattle are scarce, to the best of our knowledge there is not a single report about NTM infections in cattle in Ecuador.

Given this context, the aim of this study was to investigate the prevalence of BTB-like lesions in cattle slaughtered in abattoirs from the Ecuador’s coastal region. Additionally, we characterized mycobacterial isolates to determine the presence of MTBC or NTM species.

## Methods

### Study site

**Figure 1:**
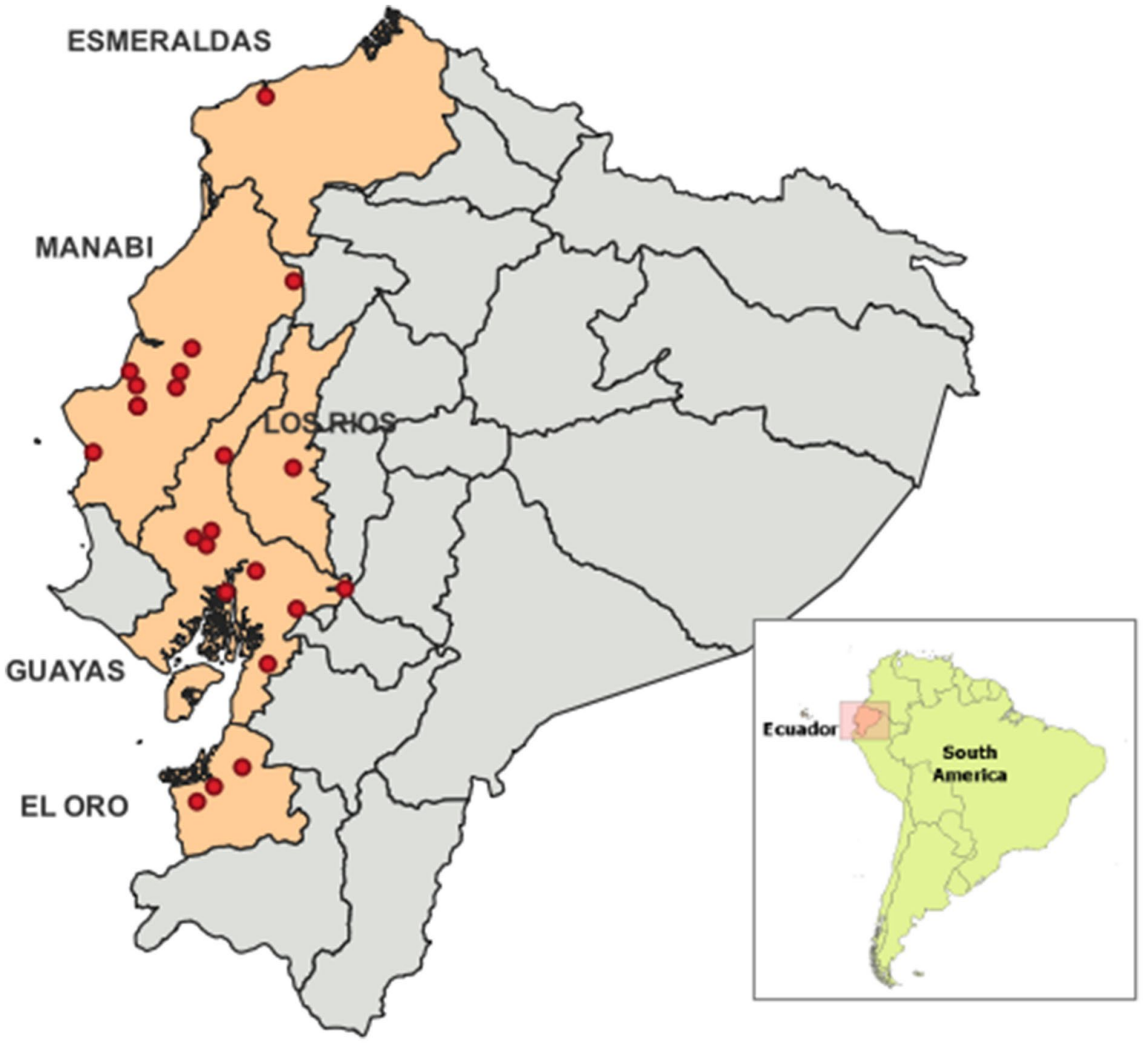
Geographycal distribution of the abbatoris included in this study within 5 provinces of the coastal region of Ecuador. The abbatoirs are identified as a red dot.

All samples were collected from 22 government-certified abattoirs across five provinces (Esmeraldas, Manabí, Los Ríos, Guayas, and Machala) located in the Ecuador’s coastal region (Figure 1) between February and November 2022.

### Sample collection

Certified veterinarians conducted post-mortem examinations of carcasses at the abattoirs to identify BTB-like lesions. A total of 5,803 bovine carcasses were examined, and tissue samples were collected from 795 carcasses showing macroscopic lesions compatible with BTB. The samples, which included submandibular, cervical, retropharyngeal, pulmonary, and mediastinal lymph nodes, were stored at −20°C until processed for *Mycobacteria* culture. As lungs are sold for human consumption in Ecuador, abattoirs did not permit sample collection from these organs. Direct smears from tissue samples or bacterial cultures were prepared and stained using the Ziehl–Neelsen method for the detection of AFB, following the guidelines for *Mycobacterium tuberculosis* laboratory diagnosis issued by the Pan American Health Organization (22).

### Mycobacteria culture

Mycobacterial cultures were performed at the BSL2 + laboratory of the National Reference Laboratory for Mycobacteria at the “Instituto Nacional de Salud Pública e Investigación” in Guayaquil, Ecuador. The cultures were prepared using the Kudoh swab method and two media types for each sample: Ogawa and Stonebrink, as previously described (23). Cultures were monitored for growth for up to 10 weeks.

### Heat inactivation and DNA isolation of suspected mycobacteria isolates

Suspected mycobacterial isolates, confirmed as AFB, were harvested from cultures and resuspended in TE buffer (10 mM Tris–HCl, 1 mM EDTA, pH 8.0). The suspensions were then heat-inactivated at 95°C for 15 min. Following inactivation, all samples were centrifuged at 10,000 g for 5 min, and the supernatants were directly used for molecular procedures, as previously described (24–27).

### Identification of MTBC and NTMs by qPCR using “VIASURE real time PCR detection kit – *M. tuberculosis* complex + non-tuberculous mycobacteria” (CerTest BIOTEC, Zaragoza, Spain)

This qualitative real-time multiplex PCR assay was designed to detect and differentiate members of the *Mycobacterium* genus, MTBC and NTM. The kit used in this study included 12 × 8-well strips, each containing lyophilized reagents necessary for the RT-PCR assay: specific primers and probes, dNTPs, buffer, polymerase, and an internal control to rule out polymerase activity inhibition. According to the manufacturer’s protocol, 15 μL of rehydration buffer was added to each well, followed by the addition of 5 μL of DNA sample to designated wells. Positive controls for MTBC and NTM (5 μL) and a negative control (5 μL) were added to separate wells, resulting in a final reaction volume of 20 μL per well. The thermal cycler (CFX96, Bio-Rad) was programmed to detect signals with a cycle threshold (Ct) ≤ 40 in the following fluorescence channels: ROX (targeting the 16S rRNA gene for *Mycobacterium* species), FAM (targeting the IS6110 and IS1081 sequences for MTBC members), and HEX (for the internal control). The PCR conditions were as follows: one initial cycle at 95°C for 2 min, followed by 45 cycles of 95°C for 10 s and 60°C for 50 s (28).

### Identification of bacterial species by matrix assisted laser desorption/ionization time of flight mass spectrometry (MALDI-ToF MS)

For sample preparation and extraction, the modified version of Bruker Daltonik’s *Mycobacteria* Extraction method version 3 (29) as described previously (30) was used. Briefly, to optimize the protein extraction, an incubation at 15 to 25°C was done following the addition of 70% formic acid and again after addition of acetonitrile. Colonies were taken from a plate and transferred to a tube containing 300 mL of HPLC grade water, then vortex to get a uniform suspension treated at 95°C for 30 min. After 5 min of cooling, 900 μL of HPLC grade ethanol was added, the suspension was centrifuged for 2 min at maximum speed, and supernatant was discarded. Centrifugation was repeated followed by removal of residual ethanol with a pipette. The pellet was left to air dry for 30 min at room temperature. Then, 50 μL of 70% formic acid was added to the pellet and mixed by pipetting for resuspension, and let for 15 min at room temperature. Then, a volume equivalent to the size of the re suspended pellet of 0.5 mm diameter zirconia/silica beads (BioSpec Products, Bartlesville, OK) were added to the suspension. The suspension was lysed using a digital disruptor genie (Scientific Industries, Inc., Bohemia, NY) set at maximum speed for 3 min. Then, 50 μL of acetonitrile was added to the lysate and mixed by pipetting, following by 5 min of incubation at room temperature. Then, the lysate was placed on the disruptor genie for 2 more min at maximum speed. The lysate was centrifuged at for 2 min at maximum speed and supernatant was collected. Finally, 1 μL of each supernatant lysate was spotted onto a ground steel target plate (Bruker Daltonik, Bremen, Germany) and air-dried for 5 min. Each spot was overlaid with 1 μL of matrix solution (a saturated solution of *α*-cyano-4-hydroxycinnamic acid in 50% acetonitrile with 2.5% trifluoroacetic acid) and air-dried for another 5 min. Spectrum acquisition was carried out on a MALDI-ToF Biotyper Microflex instrument using Flex Control 3.1 software in positive linear mode, with a laser frequency of 60 Hz and a mass range of 2,000 to 20,000 Da. Spectra were accumulated from 240 laser shots per point, and between 20 and 24 high-quality spectra were obtained for each bacterial extract.

Each ground steel target plate included a Bacterial Test Standard (BTS) for instrument calibration. As a positive control, *M. fortuitum* ATCC 6841 T was used. Mass spectral analysis was performed using the spectrum view in Flex Analysis software and MALDI-Tof Biotyper 3.1 (Bruker Daltonik GmbH, Bremen, Germany). NTM Identification was done by comparison with main spectrum profiles in the *Mycobacteria* Library version 7.0 (Bruker Daltonics, Bremen, Germany). Species identification was considered positive if the score value exceeded 2.000.

## Results

### BTB prevalence estimated by total BTB-like lesions and by AFB positive lesions

A total of 5,803 carcasses from 22 government-certified abattoirs across the provinces of Esmeraldas, Manabí, Los Ríos, Guayas, and El Oro in the Ecuador’s coastal region were inspected for BTB-like lesions in lymph nodes. The distribution of carcasses by province and abattoir is provided in Table 1. Among the inspected carcasses, 795 (13.7%; 95% CI: 12.8–14.6%) presented BTB-like lesions, that means lesions visually compatible with tuberculosis prior to laboratory confirmation. Direct smears from the lesions of these 795 tissue samples revealed that 22.4% (178/795) were positive for AFB. Considering only AFB-positive lesions, the overall presumed BTB prevalence across all carcasses was 3.1% (95% CI: 2.6–3.5%); that means assuming MTBC as the only AFB pathogen present in the lesions without molecular diagnosis confirmation.

**Table 1:** Distributions of carcasses inspected, TB-like lesions and AFB positive lesions for the 22 abbatoirs accross 5 provinces of the coastal region of Ecuador included in this study (% values of the total number of inspected carcasses).

Province/Abbatoir	Number of inspected carcasses	Number of carcasses with TB-like lesions	% carcasses with TB-like lesions (IC 95%)	Number of carcasses with AFB positive lesions	% carcasses with AFB positive lesions (IC 95%)
Total	5.803	795	13.69 (12.81–14.58)	178	3.06 (2.62–3.51)
El Oro	386	56	14.50 (2.27–28.63)	18	4.66 (2.55–6.77)
Arenillas	78	16	20.51	7	8.97
Pasaje	151	15	9.93	4	2.65
Santa Rosa	157	25	15.92	7	4.46
Esmeraldas	984	69	7.01	15	1.52 (0.75–2.29)
Esmeraldas	984	69	7.01	15	1.52
Guayas	2.578	395	15.32 (11.35–27.76)	107	4.15 (3.38–4.92)
Balzar	217	46	21.2	9	4.15
Bucay	214	23	10.75	7	3.27
Daule	216	66	30.56	25	11.57
El Triunfo	133	33	24.81	7	5.26
Guayaquil	541	50	9.24	7	1.29
Lomas de Sargentillo	123	28	22.76	6	4.88
Naranjal	561	88	15.69	31	5.53
Nobol	457	18	3.94	4	0.88
Yaguachi	116	43	37.07	11	9.48
Los Rios	132	30	22.73 (15.48–29.97)	10	7.58 (3.0–12.14)
Ventanas	132	30	22.73	10	7.58
Manabí	1723	245	14.21 (12.56–15.87)	28	1.62 (1.02–2.22)
Charapotó	200	28	14	8	4.00
Chone	229	18	7.86	2	0.87
Jipijapa	105	15	14.29	1	0.95
Portoviejo	295	20	6.78	5	1.69
24 de Mayo	17	13	76.47	2	11.76
Bolívar	177	55	31.07	3	1.69
El Carmen	324	51	15.74	1	0.31
Junín	260	21	8.07	4	1.54
Rocafuerte	116	24	20.68	2	1.72

The reported prevalence values varied across abattoirs and provinces. The highest overall presumed BTB prevalence was observed in Los Ríos province (22.7% for all lesions and 7.6% for AFB-positive lesions) and the “24 de Mayo” abattoir in Manabí province (76.5% for all lesions and 11.8% for AFB-positive lesions). Detailed prevalence data for each province and abattoir are presented in Table 1.

### Bacterial culture characterization by qPCR and MALDI-ToF MS

A total of 48 AFB isolates were recovered from tissue samples with BTB-like lesions. As detailed in Table 2, 44 of these cultures were identified as NTM using the commercial qPCR kit detailed in the methods. None of the AFB isolated cultures from cattle lesions were identified as MTBC by the discriminatory commercial qPCR kit. Additionally, four AFB cultures were either negative for NTM or MTBC.

**Table 2:** Characterization of bacterial cultures isolated from TB-like lesions by qPCR and MALDI-ToF MS (NTM means non tuberculous mycobacteria).

Sample code	Province	Abbatoir	qPCR result	Specie identification (MALDI-ToF MS)	MALDI-ToF MS score
JTLS28	Guayas	Lomas de Sargentillo	NTM	ND	ND
JTLS20	Guayas	Lomas de Sargentillo	NTM	ND	ND
JTLS24	Guayas	Lomas de Sargentillo	NTM	ND	ND
JCNA07	Guayas	Naranjal	NTM	ND	ND
JCNA15	Guayas	Naranjal	NTM	ND	ND
JCNA22	Guayas	Naranjal	NTM	ND	ND
JCNA23	Guayas	Naranjal	NTM	ND	ND
JCNA25	Guayas	Naranjal	NTM	ND	ND
JCNA26	Guayas	Naranjal	NTM	ND	ND
KGNO01	Guayas	Nobol	NTM	*Mycobacterium neoaurum*	2,02
KGNO04	Guayas	Nobol	Negative	*Nocardia* spp.	2,01
KGNO09	Guayas	Nobol	NTM	ND	ND
GMVEN03	Guayas	Ventanas	NTM	ND	ND
GMVEN14	Guayas	Ventanas	NTM	*Mycobacterium asiaticum*	2,02
GMVEN21	Guayas	Ventanas	NTM	ND	ND
GMVEN01	Guayas	Ventanas	NTM	ND	ND
GMVEN18	Guayas	Ventanas	NTM	ND	ND
GMVEN16	Guayas	Ventanas	NTM	ND	ND
GMVEN22	Guayas	Ventanas	NTM	*Mycobacterium mucogenicum*	2,08
GMVEN07	Guayas	Ventanas	Negative	*Tsukamurella paurometabola*	2,15
GMVEN12	Guayas	Ventanas	NTM	ND	ND
ITMA17	Manabi	Charapoto	NTM	ND	ND
ITMA20	Manabi	Charapoto	NTM	ND	ND
ITMA22	Manabi	Charapoto	NTM	ND	ND
ITMA23	Manabi	Charapoto	NTM	*Mycobacterium neoaurum*	2,28
ITMA24	Manabi	Charapoto	NTM	ND	ND
ITMA25	Manabi	Charapoto	NTM	ND	ND
ITMA26	Manabi	Charapoto	Negative	*Tsukamurella paurometabola*	2,24
ITMA27	Manabi	Charapoto	NTM	ND	ND
KLCA16	Manabi	El carmen	NTM	ND	ND
JLJI06	Manabi	Jipijapa	NTM	ND	ND
CZJUN13	Manabi	Junin	NTM	ND	ND
CZJUN17	Manabi	Junin	NTM	*Mycobacterium fortuitum*	2.22
CZJUN18	Manabi	Junin	NTM	ND	ND
CZJUN19	Manabi	Junin	NTM	*Mycobacterium fortuitum*	2.28
DHPOR08	Manabi	Portoviejo	NTM	ND	ND
DHPOR14	Manabi	Portoviejo	NTM	ND	ND
DHPOR16	Manabi	Portoviejo	NTM	ND	ND
DHPOR18	Manabi	Portoviejo	NTM	ND	ND
DHPOR19	Manabi	Portoviejo	NTM	*Mycobacterium neoaurum*	2.20
GGRO02	Manabi	Rocafuerte	NTM	ND	ND
GGRO14	Manabi	Rocafuerte	NTM	*Mycobacterium novocastrense*	2.21
JCBO18	Manabi	Bolivar	NTM	ND	ND
JCBO21	Manabi	Bolivar	NTM	ND	ND
JCBO22	Manabi	Bolivar	Negative	*Gordonia hongkogensis*	ND
JCBO31	Manabi	Bolivar	NTM	*Mycobacterium novocastrense*	2.17
JCBO33	Manabi	Bolivar	NTM	*Mycobacterium duvalii*	2.09
JCBO36	Manabi	Bolivar	NTM	ND	ND

ND: Means not determined.

MALDI-ToF MS analysis yielded scores above 2.000 for 14 AFB isolates, enabling species identification (Table 2). Among these, 10 isolates were identified as NTM, representing six different species: *Mycobacterium neoaureum* (3 isolates), *Mycobacterium fortuitum* (2 isolates), *Mycobacterium novocastrense* (2 isolates), *Mycobacterium asiaticum* (1 isolate), *Mycobacterium duvalii* (1 isolate), and *Mycobacterium mucogenicum* (1 isolate).

In addition, four AFB-positive cultures were identified as non-mycobacterial species: *Tsukamurella paurometabola* (2 isolates), *Nocardia* spp. (1 isolate), and *Gordonia hongkongensis* (1 isolate).

Unfortunately, many of the AFB cultures isolated could not be processed by MALDI-ToF MS and they are included in Table 2 as ND (not determined). Those isolates could not be recovered and grown after store at −80°C.

## Discussion

In this study, we conducted the first surveillance of BTB-like lesions and associated mycobacteria cultures in slaughtered cattle from the Ecuador’s coastal region. Out of 5,803 carcasses inspected across 22 abattoirs in five provinces, we observed an overall prevalence of 13.7% for lesions visually compatible with BTB, and 3.1% for lesions containing AFB. The highest prevalence of lesions at the provincial level was recorded in Los Ríos, with rates of 22.7% for overall lesions and 7.6% for lesions containing AFB. Additionally, the “24 de Mayo” abattoir in Manabí province showed alarmingly high prevalence rates of 76.5% for overall lesions and 11.8% for lesions containing AFB.

Further characterization of bacterial cultures from these lesions identified 44 NTM isolates. Out of those 44 NTM isolates, 10 of them were identified within *M. neoaureum*, *M. fortuitum*, *M. novocastrense*, *M. asiaticum*, *M. duvalii*, and *M. mucogenicum*. We call attention to the fact that no cultures of *M. bovis* or other MTBC species were detected in the lesions from the cattle included in this study. These findings suggest that opportunistic infections by NTM would contribute to lesions that look similar to tuberculosis in cattle from Ecuador. This is consistent with previous studies in Ghana and Ethiopia, where NTM were also implicated in lesions in cattle while MTBC could not be found (3, 5). Similarly, other studies in Brazil, Tanzania, and Kenya have reported both MTBC and NTM associated with lesions visually compatible with BTB in cattle and other livestock (18–21). Unfortunately, as an important limitation in our study, many of the AFB cultures isolated could not be resolved by MALDI-ToF MS (labelled as ND for not determined in Table 2). This was due to the temporal delay from the initial isolation of the AFB cultures and later characterization by MALDI-ToF MS more than one year later. Preservation of samples at −80\u00B0C was not optimal in our laboratory due to frequent blackouts. This fact affected the viability of the AFB isolates that need to be grown fresh prior to MALDI-ToF MS.

Our results suggest the potential role of opportunistic NTM infections in the etiology lesions that could be misdiagnosed as BTB by visual inspection of cattle carcasses ready for human consumption in Ecuador. However, we cannot exclude the possibility of *M. bovis* or other MTBC species infections in our study population, as BTB is present in Ecuadorian cattle (13–17). In this sense, a major limitation of this study was the inability to access pulmonary tissues at the abattoirs, as lungs are sold for human consumption; thus, only lymph nodes were examined. This fact could have reduced the probabilities of MTBC isolation, that is expected to be found in pulmonary lesions. Although problems with bacterial cultures could also explain the lack of MTBC isolates, we were able to grow MTBC reference strains as controls in every culture medium batch used in this study. Nevertheless, previous research in the Andean region of Ecuador has reported a high prevalence of BTB associated with *M. bovis* (13–17) and other colleagues have been able to isolate *M. bovis* from pulmonary lesions from cattle in Ecuador (personal communication to the authors). Therefore, the most plausible epidemiological scenario for lesions that are visually compatible with tuberculosis in cattle from Ecuador would involve infections with MTBC but also NTM, or even other AFB pathogens out of the *Mycobacterium* genus.

Our findings have significant implications for animal health and production in Ecuador. *M. bovis* is considered a major threat for cattle as the causative agent of BTB. Current surveillance and control programs are exclusively focused on *M. bovis* and BTB, and the lesions visually compatible with BTB found at abattoirs are usually considered indicative of this disease (personal communications to the authors from veterinarians carrying inspections at slaughterhouses from Ecuador). Moreover, the intradermal tuberculin skin test, prepared using tuberculin derived from *M. bovis*, is employed for BTB surveillance in Ecuador as it is recommended by the regulatory agency “Agrocalidad” from the Ministry of Agriculture. This test is mandatory to be conducted with the parallel use of tuberculin derived from *M. avium* for confirmation of reactive cattle according to “Agrocalidad” guidelines for BTB free certification, which is critical for preventing false-positive results due to infections with some NTM like *M. avium*. It is well-documented that tuberculin derived from *M. bovis* can cause cross-reactivity with NTM species, including some of those identified in this study (31, 32). In this scenario of high prevalence of NTM infections affecting cattle, it is recommended to avoid the use of single intradermal tuberculin derived from *M. bovis* test as an overestimation of BTB and underestimation of NTM infections is expected. Furthermore, visual inspections of lesions at abattoirs with or without AFB staining is definitely not accurate for BTB diagnosis due to misdiagnosis associated to NTM or other AFB infections, and must be followed by culture and molecular characterization of the isolated pathogens.

The presence of NTM in carcasses prepared for human consumption in abattoirs in Ecuador would be a potential threat for public health that deserves further research. Although NTM are generally considered non contagious opportunistic pathogens, some species like *M. avium* complex are zoonotic. Moreover, opportunistic NTM infections in humans are recognized as an emerging public health issue in Ecuador and worldwide, but the transmission dynamics remain unclear; although most of NTM infections have been associated with environmental exposure, the potential role of animal reservoirs deserves further investigation (33, 34). In this sense, our results suggest a potential source of opportunistic NTM infections in humans linked to occupational risk for veterinarians and other abattoir workers, or food borne transmission from infected meat.

Finally, we draw attention to the fact that, in addition to NTM, other AFB opportunistic pathogens outside the *Mycobacterium* genus were identified such as *Tsukamurella*, *Nocardia*, and *Gordonia* (35). Notably, a recent study conducted in Venezuela identified these same three genera of AFB bacteria, which were initially misdiagnosed as NTM in human patients (34). These findings underscore the importance of incorporating molecular tools, such as qPCR and MALDI-ToF MS, for accurate identification of MTBC, NTM and other AFB genera in humans and animals.

In conclusion, NTM and other AFB were found in lesions in slaughtered cattle from the Ecuador’s coastal region and represent a source of opportunistic infections that could be misdiagnosed as BTB. More comprehensive cattle surveillance and control programs that integrate molecular tools for the precise identification of etiological agents of lesions and disease should be implemented in Ecuador and other regions.

## Data Availability

The original contributions presented in the study are included in the article/supplementary material, further inquiries can be directed to the corresponding author.
